# *Salmonella* Biofilm Formation under Fluidic Shear Stress on Different Surface Materials

**DOI:** 10.3390/foods12091918

**Published:** 2023-05-08

**Authors:** Hudson T. Thames, Diksha Pokhrel, Emma Willis, Orion Rivers, Thu T. N. Dinh, Li Zhang, Mark W. Schilling, Reshma Ramachandran, Shecoya White, Anuraj T. Sukumaran

**Affiliations:** 1 Department of Poultry Science, Mississippi State University, Starkville, MS 39762, USA; htt37@msstate.edu (H.T.T.);; 2 Institute for Imaging & Analytical Technologies, Mississippi State University, Starkville, MS 39762, USA; 3 Tyson Foods, 2200 W. Don Tyson Parkway, Springdale, AR 72762, USA; 4 Department of Food Science, Nutrition, and Health Promotion, Mississippi State University, Starkville, MS 39762, USA

**Keywords:** *Salmonella*, biofilm, crystal violet, shear stress, poultry processing

## Abstract

This study characterized biofilm formation of various *Salmonella* strains on common processing plant surface materials (stainless steel, concrete, rubber, polyethylene) under static and fluidic shear stress conditions. Surface-coupons were immersed in well-plates containing 1 mL of *Salmonella* (6 log CFU/mL) and incubated aerobically for 48 h at 37 °C in static or shear stress conditions. Biofilm density was determined using crystal violet assay, and biofilm cells were enumerated by plating on tryptic soy agar plates. Biofilms were visualized using scanning electron microscopy. Data were analyzed by SAS 9.4 at a significance level of 0.05. A surface–incubation condition interaction was observed for biofilm density (*p* < 0.001). On stainless steel, the OD_600_ was higher under shear stress than static incubation; whereas, on polyethylene, the OD_600_ was higher under static condition. Enumeration revealed surface–incubation condition (*p* = 0.024) and surface–strain (*p* < 0.001) interactions. Among all surface–incubation condition combinations, the biofilm cells were highest on polyethylene under fluidic shear stress (6.4 log/coupon; *p* < 0.001). Biofilms of *S.* Kentucky on polyethylene had the highest number of cells (7.80 log/coupon) compared to all other strain–surface combinations (*p* < 0.001). Electron microscopy revealed morphological and extracellular matrix differences between surfaces. Results indicate that *Salmonella* biofilm formation is influenced by serotype, surface, and fluidic shear stress.

## 1. Introduction

*Salmonella* is one of the most persistent pathogens in the poultry industry. Every year, greater than 1 million *Salmonella* infections occur in the U.S., with poultry responsible for a significant portion of these infections [1,2]. Poultry are asymptomatic carriers of *Salmonella* which are highly transmissible throughout the flock once they are introduced [3]. *Salmonella* is introduced through a variety of vectors, including as wild animals, insects, and rodents, as well as through personnel [4,5]. These contaminated birds then introduce *Salmonella* to processing plants and food contact surfaces. The continuous evolution of *Salmonella* is one of the many contributing factors towards its persistence in poultry and can be attributed to the development of over 2500 serotypes as classified by the World Health Organization.

While there are more than 2500 *Salmonella* serotypes, less than 100 cause foodborne infection. *S.* Typhimurium, *S.* Enteritidis, and *S.* Heidelberg are some of the most commonly isolated serotypes [6]. However, in recent years, studies have indicated a stronger prevalence of strains such as *S.* Schwarzengrund, *S.* Kentucky, and *S.* Infantis [7,8,9,10]. Another serotype which recently made headlines is *S.* Reading, which was responsible for a multistate outbreak in turkey meat, infecting 356 individuals and hospitalizing 132 [11]. A surge in new dominant serotypes infers that there are changes in *Salmonella* survival mechanics which could be contributing to persistence. Studies over the past several years have indicated that biofilm formation is one of the primary survival mechanisms of *Salmonella* [12,13]. 

Biofilms are complex communities of bacteria irreversibly attached to a surface, living in organized structures that are not normally found in planktonic cultures [13,14]. This intricate cell network is the predominant method of bacterial growth in the environment and is frequently responsible for many infections and outbreaks. It has been estimated that biofilms are responsible for nearly 80% of bacterial infections [15]. Biofilms are unique from planktonic cells in that they contain various extracellular components such as cellulose, curli fimbriae, unique proteins, and various polysaccharides [15]. In nature, diverse communities of cells in biofilms have an enhanced ability for gene transfer which may contribute to the acquisition of new genes for survival and increased virulence [13]. This enhanced ability to survive is particularly concerning for poultry production, as it may increase the risk of *Salmonella* exposure to birds. 

While biofilms can form on broiler meat, they are more often found on various abiotic surfaces such as stainless steel, concrete, plastic, and glass [15]. A number of studies have found that *Salmonella* biofilms are much more resistant to commercial sanitizers such as chlorine and quaternary ammonium than planktonic cells [16,17]. This is especially challenging for sanitation in poultry processing, as these compounds are most often utilized in commercial processing plants. Particularly, stainless steel is the primary alloy of most processing equipment, chill tanks, and wall surfaces. In most plants, concrete is used on the processing floor. Ultra-high molecular weight polyethylene is used on deboning tables, on cutlery handles, and in some assembly mechanisms. Although not specifically used on the processing floor, glass is a transparent material used in processing facilities and other aspects of the food industry. Given that *Salmonella* biofilms have been observed on different materials, it is important to investigate the role of surface material in biofilm formation, as the surface material may affect biofilm matrix density and cell attachment. However, currently, there are limited data on the growth characteristics of different serotypes of *Salmonella* on the various food contact surfaces that are utilized in poultry processing. With this in mind, certain surfaces may provide greater support for biofilm formation, thus increasing the risk of contamination by continued shedding of viable *Salmonella* cells. 

A multitude of studies have attempted to characterize *Salmonella* biofilms. However, the diversity of analytical tools and inconsistencies in methodology makes this challenging [18,19]. One of the most common biofilm detection methods is the crystal violet assay that uses a polystyrene well plate. However, even for this one technique, there are a variety of ways that the methodology is applied, which leads to challenges in comparing results [20]. Additionally, while well plates have been used as the sole surface for attachment in the majority of studies for determining biofilm characteristics, environmental factors such as surface material and fluidic shear stress may affect biofilm formation. Shear stress is of particular interest in poultry processing as it describes the friction caused by fluid moving over a solid surface. Processing plants have a continuous flow of water in chill tanks, dip tanks, and spray cabinets that drains across processing surfaces. Modifying existing biofilm detection methods to account for this environmental factor may more accurately simulate biofilm growth in commercial settings [21]. 

Therefore, the objective of this study was to determine the effect of surface materials and fluidic shear stress on the biofilm-forming ability of different serotypes of *Salmonella* using a modified crystal violet assay, enumeration by direct plating, and scanning electron microscopy.

## 2. Materials and Methods

### 2.1. Salmonella Strains

Five *Salmonella* strains were used in this study: *S.* Typhimurium ATCC 14028, *S.* Enteritidis ATCC 4931, *S.* Reading turkey outbreak strain 0330, reference strain 0326, and *S.* Kentucky. *S.* Typhimurium and *S.* Enteritidis were acquired from a previous study. Both strains of *S.* Reading were provided by Dr. Timothy Johnson, University of Minnesota. *S.* Kentucky was previously collected from a commercial processing plant and serotyped by the National Veterinary Services Laboratories, Ames, Iowa.

### 2.2. Surface Materials

Four surface materials were analyzed in this study: stainless steel (RD128-316), concrete (RD128-CC), ethylene propylene diene monomer (EPDM) rubber (RD128-EPDM), and ultra-high molecular weight (UHMW) polyethylene (RD128-PE). Coupons made of each surface material were purchased from Biosurface Technologies Corporation (Bozeman, Montana). Each coupon was 12.7 mm in diameter and 3.8 mm thick. Each of the four surface materials was selected based on its use in poultry processing plants.

### 2.3. Bacterial Suspension Preparation

Stock cultures of each strain of *Salmonella* were stored at −80 °C in 20% glycerol. Each strain was cultured three times on XLT4 to maximize the viability and purity of each strain. Working cultures were prepared by incubating an individual colony in 10 mL of tryptic soy broth (TSB) at 37 °C for 18–20 h and serially diluting each strain to 6.00 log CFU/mL. For each replication, new working cultures of all five strains were prepared. 

### 2.4. Crystal Violet Assay

A modified crystal violet assay was established based on several previously described protocols [22,23,24,25]. Coupons of each surface material were cleaned by scrubbing with soap and water and were then autoclaved at 121 °C for 15 min. Coupons were then placed in 24-well tissue culture plates using sterile forceps. One milliliter of prepared inoculum (~10^6^ CFU/mL) in TSB and negative controls containing only TSB were then pipetted into individual wells containing the coupons. Well plates designated for static conditions were incubated at 37 °C for 48 h without shaking. Samples designated for fluidic shear stress conditions were incubated in an incubator shaker (New Brunswick™ Scientific Excella^®^ E24, Enfield, CT, USA) rotating at 150 rpm for 48 h at 37 °C to simulate the turbulent flow of water movement *Salmonella* may be exposed to in a processing facility. After incubation, coupons were removed from the solution using sterile forceps and gently rinsed with sterile deionized water to remove loosely attached cells. The coupons were then placed in new sterile 24-well plates containing 1 mL of crystal violet solution and incubated at room temperature for 45 min (0.41% *w*/*v* dye, AC447570500, ACROS Organics). After incubation, the coupons were removed from the wells with sterile forceps and gently rinsed again with sterile deionized water. The coupons were placed in a final 24-well plate and 1 mL of PROTOCOL^®^ decolorizing solution (80% isopropyl alcohol, 20% acetone) was pipetted into each well. The well plate was then incubated at room temperature for 45 min to allow enough time for the bound crystal violet to be removed from the coupons. After incubation, 200 µL of the dissolved solution from each well was pipetted into a 96-well plate and the optical density (OD_600_) was measured using a spectrophotometer (Cytation 1, BioTek Inc., Winooski, VT, USA).

### 2.5. Enumeration of Biofilm Attached Cells

For the enumeration of cells present in biofilms, coupons were prepared similarly to the crystal violet assay. One milliliter of prepared inoculum (~10^6^ CFU/mL) in TSB and negative controls containing only TSB were pipetted into wells containing the coupons, and the well plates were incubated in static and shear stress conditions as before. After incubation, the coupons were removed from the well plates with sterile forceps and gently rinsed with sterile deionized water to remove planktonic cells. Each coupon was placed in a 12 mL snap cap tube, which contained 10 mL of sterile TSB, and the tubes were vortexed for 1 min to remove the attached biofilm from the coupon surface. Serial dilutions were then prepared using TSB, and the cells were enumerated on tryptic soy agar media plates (TSA) using the spread plate method and incubated for 24 h at 37 °C. 

### 2.6. Scanning Electron Microscopy

Scanning electron microscopy (SEM) was performed to verify the attachment of cells and visualize the formation of a biofilm extracellular matrix. For each experimental replication, three additional coupons that were inoculated with *S.* Reading outbreak strain 0330 were incubated in static and fluidic shear stress conditions and prepared for SEM. The coupons were removed from their respective incubation conditions with sterile forceps and gently rinsed with sterile deionized water to remove loosely attached planktonic cells. The coupons were placed in a new 24-well plate and 1 mL of fixative (2.5 glutaraldehyde, 2% paraformaldehyde, 0.1 M sodium cacodylate) was pipetted into each well. The coupons were incubated at room temperature (include temperature here) for 45 min in the fixative and then placed in a new sterile 24-well plate to dry. Each coupon was then sputter coated with 30 µg of platinum and imaged using a JEOL JSM-6500F Field Emission Scanning Electron Microscope. 

### 2.7. Statistical Analysis

This study was a completely randomized design with a factorial arrangement of five salmonella strains, four surface materials, and two incubation conditions with a total of three replications. For the crystal violet assay, differences in the degree of biofilm formation (biofilm matrix density) between incubation conditions and strains on each surface were examined within the GLIMMIX procedure of SAS version 9.4 using the relative O.D. values which were obtained by subtracting the O.D. values of each treatment from the negative controls. Means were separated by the LS means procedure at a *p* value of <0.05. Differences in the actual number of biofilm-attached cells produced from each strain were also determined using SAS version 9.4 within the GLIMMIX procedure and means were separated at a *p* value of <0.05.

## 3. Results

### 3.1. Crystal Violet Assay

**Figure 1:**
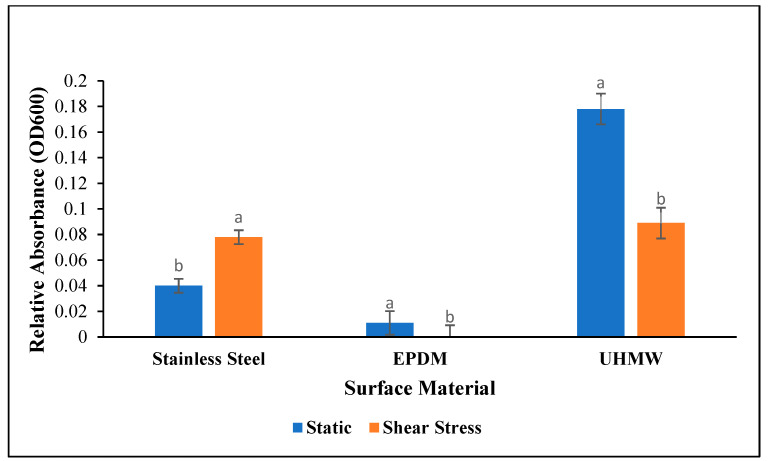
The effect of surface and incubation condition on *Salmonella* biofilm formation on stainless steel, EPDM rubber, and UHMW polyethylene (*p* = 0.013). The relative optical density of the treatments was obtained by subtracting the negative controls from the treatment averages. ^a,b^ Means within each surface without similar letters differ significantly.

An interaction between surface material and incubation condition affected biofilm extracellular matrix density (*p* < 0.001). On stainless steel, the relative absorbance of *Salmonella* under shear stress was nearly double that of static incubation at an absorbance of 0.079 and 0.041, respectively (Figure 1). The relative absorbance on EPDM under static incubation and shear stress was 0.011 and 0.00, respectively, meaning the OD_600_ was not significantly different from the negative controls. The concrete coupons were porous, which made it impossible to remove excess crystal violet. As a result, the optical density for all of the concrete treatments was above detectable limits and quantifiable values were not obtained. On UHMW polyethylene, the relative absorbance was greater in static incubation than with shear stress at an average absorbance of 0.178 and 0.089, respectively (*p* < 0.001). While the relative optical densities were used for statistical analysis, the OD_600_ from each treatment can be seen in the Supplementary Table S1.

### 3.2. Enumeration of Biofilm Attached Cells

**Figure 2:**
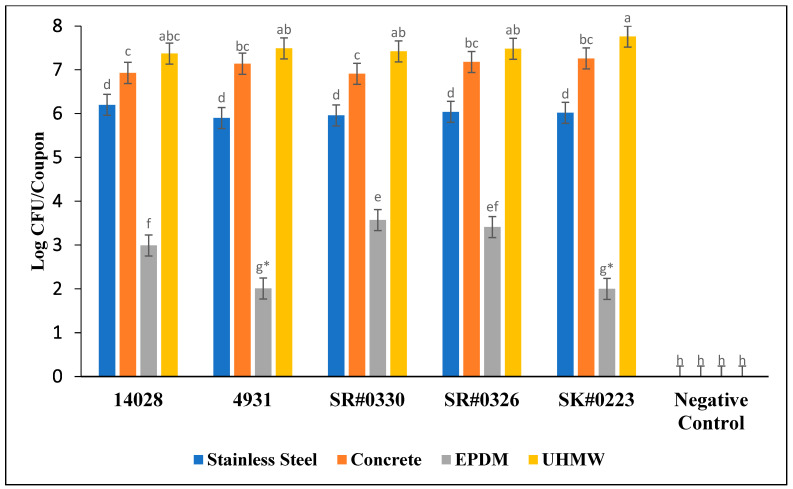
The effect of surface type on the number of attached cells for each strain (*p* < 0.001). (*) indicates that treatments were below the limit of detection of 2.00 log CFU/coupon. ^a–h^ Means without similar letters differ significantly.

There was a surface material and strain interaction (*p* < 0.001) in the number of biofilm-attached cells (Figure 2). However, differences between strains were only observed on EPDM rubber, which exhibited little evidence of biofilm formation. Considering this, significant differences were observed between surface materials (*p* < 0.001). The average number of attached cells on each surface irrespective of strain was as follows: stainless steel: 6.02 log CFU/coupon, concrete: 7.04 log CFU/coupon, EPDM rubber: 2.79 log CFU/coupon, UHMW polyethylene: 7.50 log CFU/coupon. When averaged across all strains, the average number of cells attached to concrete and UHMW were greater than on stainless steel by 1.00 and 1.50 log CFU/coupon, respectively (*p* < 0.001). For each treatment and replication, no colonies were detected from negative controls. 

### 3.3. Scanning Electron Microscopy

**Figure 3:**
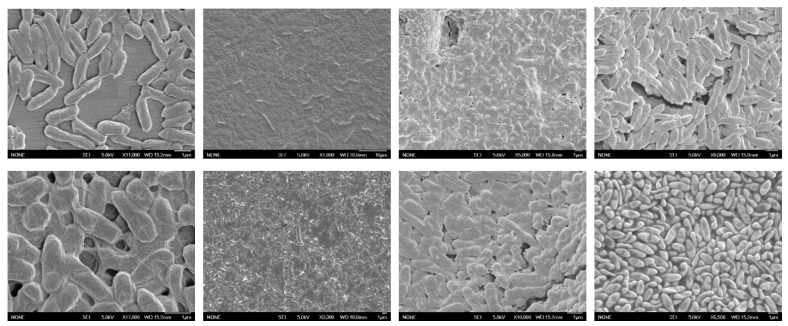
SEM images of *S.* Reading outbreak strain on all four surface materials in both static and shear stress incubation conditions. The surface materials from left to right are as follows: stainless steel, EPDM rubber, concrete, UHMW polyethylene. The top row is static incubation, and the bottom row is under fluidic shear stress.

Representative SEM images of the outbreak strain of *S.* Reading are shown in Figure 3. In the first column, a monolayer of cells can be seen under static incubation with a rudimentary biofilm extracellular matrix. The cells are firmly attached to the surface and attached to one another. However, under shear stress, the biofilm matrix is much more complex. Cells are organized in multiple layers and extracellular biofilm components are clearly visible. In the second column, a small number of scattered attached cells are visible on rubber, both in static incubation and under shear stress. Morphological differences between incubation conditions on concrete can be observed in column three. The biofilm density of *Salmonella* on concrete is denser than for other treatments. In column four, morphological differences can be observed between *S.* Reading on UHMW in static and shear stress conditions. The matrix structure in static conditions more closely resembles stainless steel and concrete under shear stress. However, multilayered structures are clearly visible in both conditions.

## 4. Discussion

### 4.1. Biofilm Density

Of the existing literature, there are very few studies that use a crystal violet assay on surfaces other than polystyrene well plates. One study which clearly states data interpretation similar to that of this study reported optical densities on polystyrene that were similar to those of the polyethylene that was used in this study [25]. In both this study and that conducted by Obe et al. (2021), *S.* Kentucky and Enteritidis exhibited biofilm-forming abilities on plastic. However, the same control strain of *S.* Typhimurium (ATCC 14028) exhibited the same biofilm-forming potential as compared to the other strains in this study, whereas it was considered weak in the study by Obe et al. (2021). This may be attributed to the use of polyethylene coupons in this study which could allow for improved attachment. One additional study utilized crystal violet staining on stainless steel as a visual indicator rather than a means to quantify biomass density [26]. Purple-stained biofilms were visible on stainless steel in our study, which was similar to this study. Interestingly, fluidic shear stress influenced the density of the biofilm extracellular matrix, without affecting the average number of attached cells. While the literature is limited on this environmental effect, it has been reported that shear stress can improve oxygenation and enhance the expression of biofilm components [27]. It is possible that the improved oxygenation allowed for sustained biofilm matrix production on stainless steel. However, the effect of shear stress on biofilm density was dependent on the surface material in this study. By contrast, shear stress significantly hindered the production of biofilm components on polyethylene. While shear stress may enhance biofilm synthesis, it has been noted that in some cases, hydrodynamic forces could slough off the outer layers of the biofilm matrix that are not as well attached [27]. This would explain the differences observed in Figure 3 on polyethylene. 

As pointed out by other authors, CV is a viable tool for a limited number of surfaces [28]. It was observed that CV was not suitable for use on concrete in this study, given that excessive retention resulted in results above detectable limits. While still measurable, the rubber coupons in general had a higher retention of crystal violet as compared to stainless steel and polyethylene. In each of the conditions, the CV was not rinsed as easily with water as compared to stainless steel and polyethylene. However, enumeration and SEM demonstrated that despite the higher OD_600_, there were significantly less cells attached. Regardless, while other methods are able to monitor biofilm formation on more surfaces, crystal violet assays are more affordable and less time consuming, all while requiring a minimal amount of equipment. By combining aspects of other methods, such as the use of biofilm reactor coupons, with the methodology of crystal violet assays, a simplistic method can be developed that more accurately represents biofilm growth on processing surfaces. However, one shortcoming of the crystal violet assay is the lack of standardization in classifying biofilm strength. Categorizing biofilm-forming strength based on optical densities is reliant on several key factors, which include the optical densities of the negative controls, the blank used, whether or not a well plate lid is used, as well as other environmental factors. Most of the existing studies do not clarify these details which can affect how the data are presented. Thus, categorizing the biofilms as “weak, moderate, or strong” was not used in this study.

### 4.2. Enumeration of Biofilm Attached Cells

While it was not possible to directly compare the results of the crystal violet assays in this study to the previous literature, the number of attached cells in this study were similar to previous findings. After 48 h of incubation, biofilm attached cells of *S.* Typhimurium, Enteritidis, and Agona on stainless steel were 5.78 and 6.60 log CFU/coupon, respectively [23,29]. These values are similar to the 6.02 log CFU/coupon that was quantified in the current study. On concrete, differences of less than 1.00 log were observed between studies. Corcoran and Joseph reported counts of 6.81 and 6.50 log CFU/coupon, respectively [16,23]. Interestingly, counts on rubber were less in this study when compared to previous findings. While there are virtually no other publications using the same brand of rubber coupons, Dygico and Sadekuzzaman reported counts of 4.80 and 6.00 log CFU/coupon, respectively [28,30]. Whereas, in this study, the average number of cells was much lower at 2.79 log CFU/coupon. A number of surface factors can affect cell attachment, including hydrophobicity, surface coating, or roughness of the surface. While cell attachment in this study was similar to previous findings with regards to stainless steel, concrete, and polyethylene, it is possible that one of these environmental factors inhibited *Salmonella* attachment on rubber coupons [31]. However, despite repeated uses and multiple test trials, the number of attached cells on EPDM rubber remained substantially less than on the other surfaces. It is also possible that this brand of rubber has a unique surface coating that hindered bacterial attachment. Biofilm attached cell counts on polyethylene were similar to some previous findings. Two studies reported 7.50 to 7.60 log CFU/coupon of *Salmonella* attachment on polyethylene [16,32]. Findings in this study were very consistent with previous investigations of polyethylene. 

### 4.3. Scanning Electron Microscopy

The outbreak strain of *S.* Reading #0330 was the only strain used for imaging. This decision was made primarily due to the fact that in trials, there were no major visible differences between strains, indicating that the images of other strains were very similar. This specific strain was also selected due to its recent isolation from turkey meat. Very few studies have investigated this outbreak strain beyond basic antimicrobial testing, and it was hypothesized that acquiring images of this particular strain could provide insight into its survival mechanism. As the intent was to view the overall structure of the biofilm, the sample preparation was non-invasive, incorporating short fixation/dehydration times. This preparation proved to be effective in preserving the extracellular structure; thus, it may contribute to some of the visual differences between this study and previous research. For example, in this study, the complexity of biofilm matrices on stainless steel was clearly visible in both static and shear stress conditions, whereas another study found less of an established extracellular matrix even after 5 days of incubation on stainless steel [33]. Given that the number of biofilm-attached cells were similar in this study to the one conducted by Wang et al., the sample preparation in this study may help preserve the extracellular matrix as seen in the images from this study. Interestingly, on rubber, there was little evidence of a biofilm in either condition, which contradicts what has been reported by several previous studies [30,31,32]. In all three of these studies, layers of attached *Salmonella* cells are clearly visible with various extracellular components. As discussed previously, there are a number of factors which could contribute to the lack of cell attachment on the specific brand of rubber coupon that was used in this study. However, more research is necessary to understand why this phenomenon occurred. While there were no optical densities recorded on concrete, the SEM images clearly indicate a well-formed biofilm in both conditions. Concrete contains many pores and crevices which allow bacteria more opportunities for attachment. This is evident by the layers of cells and the matrix components in the two images. However, porous concrete proved to be more difficult to dry than the other materials prior to sputter coating. To achieve the appropriate dryness, it is recommended to dry coupons in a vacuum chamber if available. Images in this study of *Salmonella* on polyethylene were similar to some previous findings reported by [32]. In both studies, multiple layers of cells are clearly visible with a surplus of extracellular components. 

## 5. Conclusions

All five strains of *Salmonella* demonstrated the ability to form biofilms on stainless steel, concrete, and polyethylene. While fluidic shear stress did not affect the number of attached cells, it did affect the biofilm matrix density. Interestingly, this effect was dependent upon the surface material. Fluidic shear stress enhanced the biofilm-forming ability of *Salmonella* on stainless steel, whereas on polyethylene, it hindered biomass density. Overall, the highest number of cells was found on polyethylene, and the most developed biofilm matrix was observed on polyethylene in static conditions. While no optical densities were recorded, SEM images indicated that the biofilm density on concrete was similar to that of polyethylene. Therefore, stainless steel, polyethylene, and concrete present the highest risk for *Salmonella* biofilm formation based on the metrics used in this study. 

While a limited number of surface materials were used in this study, based on these results, it would be worthwhile investigating the biofilm-forming ability of *Salmonella* on additional metallic alloys and coated surfaces. Although less commonly explored in poultry, aluminum and copper are utilized in various food preparation facilities and wastewater management. Furthermore, there are various steel alloys available as coupons for investigation. Based on these findings, it is possible one of these variants could offer greater resistance to biofilm formation. While not used in this study, coated concrete is another treated material worth exploring, as this treatment accounts for the porous nature of concrete and could inhibit the attachment of *Salmonella*. Moving forward, additional research is needed to understand why these differences were observed. While these results provide valuable insight into what happens when *Salmonella* is exposed to different environments, more advanced molecular techniques could explain why biofilm formation varies between these conditions.

## Data Availability

The data presented in this study are available on request from the corresponding author.

## Supplementary Materials

The following supporting information can be downloaded at: https://www.mdpi.com/article/10.3390/foods12091918/s1, Table S1: The OD_600_ of each treatment combination before normalization by subtracting the controls.
