# Supplementary material for: Salmonella Biofilm Formation under Fluidic Shear Stress on Different Surface Materials
Source: Foods. 2023 May 8;12(9):1918. doi: 10.3390/foods12091918 (PMC10178852; doi:10.3390/foods12091918)
Supplement: Supplementary file 1 [file foods-12-01918-s001.zip › foods-2350055-supplementary.pdf]

Table S1. The OD_{600} of each treatment combination before normalization by subtracting the controls.

Surface	Incubation	Strain	OD_{600}
Stainless Steel	Static	14028	0.081
Stainless Steel	Static	4931	0.079
Stainless Steel	Static	SR#0330	0.078
Stainless Steel	Static	SR#0326	0.076
Stainless Steel	Static	K0223	0.094
Stainless Steel	Static	Control	0.041
Stainless Steel	Shear Stress	14028	0.100
Stainless Steel	Shear Stress	4931	0.086
Stainless Steel	Shear Stress	SR#0330	0.122
Stainless Steel	Shear Stress	SR#0326	0.113
Stainless Steel	Shear Stress	K0223	0.099
Stainless Steel	Shear Stress	Control	0.025
Rubber	Static	14028	0.224
Rubber	Static	4931	0.226
Rubber	Static	SR#0330	0.226
Rubber	Static	SR#0326	0.253
Rubber	Static	K0223	0.230
Rubber	Static	Control	0.220
Rubber	Shear Stress	14028	0.087
Rubber	Shear Stress	4931	0.120
Rubber	Shear Stress	SR#0330	0.085
Rubber	Shear Stress	SR#0326	0.150
Rubber	Shear Stress	K0223	0.105
Rubber	Shear Stress	Control	0.133
Polyethylene	Static	14028	0.166
Polyethylene	Static	4931	0.186
Polyethylene	Static	SR#0330	0.244
Polyethylene	Static	SR#0326	0.232
Polyethylene	Static	K0223	0.196
Polyethylene	Static	Control	0.027
Polyethylene	Shear Stress	14028	0.083
Polyethylene	Shear Stress	4931	0.069
Polyethylene	Shear Stress	SR#0330	0.078
Polyethylene	Shear Stress	SR#0326	0.111
Polyethylene	Shear Stress	K0223	0.198
Polyethylene	Shear Stress	Control	0.018
